# Optimizing academic engagement and mental health through AI: an experimental study on LLM integration in higher education

**DOI:** 10.3389/fpsyg.2025.1641212

**Published:** 2025-10-01

**Authors:** Min Zhang

**Affiliations:** College of Humanities and Arts, Xi'an International University, Xi‘an Shaanxi, China

**Keywords:** Large Language Models, academic writing, mental health, student engagement, higher education, guided instruction, educational technology, AI-assisted learning

## Abstract

**Background:**

In alignment with UNESCO's Sustainable Development Goal 4 (SDG4), which advocates for inclusive and equitable quality education, the integration of Artificial Intelligence tools—particularly Large Language Models (LLMs)—presents promising opportunities for transforming higher education. Despite this potential, empirical research remains scarce regarding the effects of LLM use on students' academic performance, mental well-being, and engagement, especially across different modes of implementation.

**Objective:**

This experimental study investigated whether a guided, pedagogically grounded use of LLMs enhances students' academic writing quality, perceived mental health, and academic engagement more effectively than either unguided use or no exposure to LLMs. The study contributes to UNESCO's “Futures of Education” vision by exploring how structured AI use may foster more inclusive and empowering learning environments.

**Method:**

A total of 246 undergraduate students were randomly assigned to one of three conditions: guided LLM use, unguided LLM use, or a control group with no LLM access. Participants completed a critical writing task and standardized instruments measuring academic engagement and mental well-being. Prior academic achievement was controlled for, and writing quality was assessed using Grammarly for Education.

**Results:**

Students in the guided LLM condition achieved significantly higher scores in writing quality and academic engagement compared to the control group, with large and moderate effect sizes, respectively. Modest improvements in mental health indicators were also observed. By contrast, unguided use yielded moderate gains in writing quality but did not produce significant effects on engagement or well-being.

**Conclusion:**

The findings highlight the critical role of intentional instructional design in the educational integration of AI tools. Structured guidance not only optimizes academic outcomes but also supports students' wellbeing and inclusion. This study offers empirical evidence to inform ongoing debates on how digital innovation can contribute to reducing educational disparities and advancing equitable learning in the post-pandemic era.

## 1 Introduction

The accelerated integration of Artificial Intelligence (AI) into higher education is reshaping the academic landscape at an unprecedented pace. In particular, the emergence of Large Language Models (LLMs), such as OpenAI's ChatGPT, has generated both enthusiasm and apprehension among educators and policymakers. While some institutions have embraced these technologies as tools to enhance personalization, accessibility, and innovation in teaching, others have expressed concern about academic integrity, student dependency, and the erosion of critical thinking. The current moment thus presents a pivotal opportunity—and challenge—for universities to evaluate the pedagogical value of LLMs and their broader impact on student learning (Sharma et al., 2025).

The urgency of this evaluation is underscored by the widespread and rapid adoption of LLMs in academic contexts. For instance, recent headlines such as “More than half of UK undergraduates say they use AI to help with essays” (Adams, 2024) reflect a shift in student practices that institutions are still struggling to regulate or harness effectively (Fritz et al., 2024). Despite this proliferation, empirical evidence remains limited, especially regarding how different modalities of LLM implementation—guided versus unguided use—affect students' academic performance, mental wellbeing, and engagement. Given the scale and speed of adoption, addressing this gap has become an urgent priority for educators and researchers alike.

In this context, international policy frameworks such as UNESCO's Sustainable Development Goal 4 (SDG4), which promotes inclusive, equitable, and quality education, and the “Futures of Education” initiative offer critical guidance. The latter calls for reimagining how knowledge is produced, valued, and shared, with a strong emphasis on human-centered, ethically grounded digital innovation. This vision aligns closely with the need to understand how emerging technologies like LLMs can support not only academic excellence, but also psychological wellbeing and inclusive engagement among students.

Integrating AI into university education is not merely a matter of technological adaptation; it compels a re-examination of core pedagogical processes. Academic writing, for example, remains a central yet often stressful academic demand, both difficult to master and to assess objectively (Ayeni et al., 2024). Simultaneously, student mental health has emerged as a pressing concern in higher education, particularly within competitive and international environments (Molodynski et al., 2021). Academic engagement—the emotional, cognitive, and behavioral investment in learning—is equally critical, yet sensitive to instructional design and motivation (Lin, 2024).

Although interest in educational applications of LLMs is growing (Ng et al., 2024), including recent efforts to synthesize their contributions to personalized learning (Sharma et al., 2025), few studies have experimentally assessed their impact on these three domains within controlled settings (Jungherr, 2023). Furthermore, how these tools are introduced—whether with structured guidance or left to student discretion—may significantly influence their effectiveness and students' emotional and cognitive responses to academic tasks (Chang, 2024).

The present study addresses this research gap by experimentally examining the effects of guided versus unguided use of an LLM on undergraduate students' academic writing quality, perceived mental health, and academic engagement. Conducted in an international university in China, the study employed a standardized writing task and randomized group assignment (guided use, unguided use, control) to determine whether structured integration enhances learning outcomes and wellbeing. The results aim to inform evidence-based, ethical practices for AI integration in higher education and contribute to global discussions on how digital tools can advance more inclusive, resilient, and human-centered academic environments.

## 2 Theoretical framework and empirical background

### 2.1 LLMs in higher education

LLMs, such as GPT-4, are increasingly present in higher education as tools to assist with language production, research synthesis, and academic writing (Lu et al., 2024). Their growing use among university students has sparked institutional interest in understanding how these tools influence learning outcomes. However, emerging evidence suggests that the pedagogical value of LLMs depends less on their availability than on the instructional design that accompanies their use (Robleto et al., 2024).

A useful framework for analyzing the educational integration of technology is the Technological Pedagogical Content Knowledge (TPACK) model developed by (Mishra and Koehler 2006). TPACK posits that effective technology-enhanced instruction requires the intersection of three types of knowledge: disciplinary content, pedagogical strategies, and technological tools. In this model, the mere introduction of digital resources does not guarantee meaningful learning. Rather, it is the thoughtful alignment of those tools with pedagogical goals and disciplinary content that fosters deep understanding and transferable skills.

This framework is particularly relevant to the use of LLMs. In a guided implementation, students receive explicit instructions on how to use the model to support key aspects of academic writing—such as developing argument structure, paraphrasing source material, or revising according to disciplinary conventions (Yan et al., 2024). This structured use of the tool reflects the TPACK ideal: technology embedded within a coherent pedagogical plan.

By contrast, unguided use of LLMs lacks this intentional alignment. Although students may independently explore the tool's capabilities, they do so without pedagogical framing, which may result in inconsistent outcomes. Unguided users might underuse the tool, rely on it uncritically, or fail to recognize its limitations (Wang, 2022). Finally, students in the control group, with no access to LLMs, must rely entirely on their prior writing skills and internal resources. While this condition mirrors traditional academic expectations, it may pose additional cognitive and emotional challenges for students with lower confidence or weaker academic preparation (Ayeni et al., 2024). Building on this theoretical foundation, the present study investigates how different instructional approaches to LLM use—guided, unguided, or absent—affect academic writing, mental well-being, and engagement. The TPACK framework supports the hypothesis that pedagogically framed LLM use will yield superior outcomes across all domains.

## 3 Academic writing quality

Academic writing is a core component of higher education, particularly in the humanities and social sciences. It demands clarity of argument, mastery of disciplinary conventions, and grammatical precision—competencies that students often struggle to develop and instructors find difficult to evaluate objectively (Ayeni et al., 2024). Studies have shown that structured instructional approaches, such as modeling, scaffolding, and feedback, consistently improve students' writing skills (De La Paz, 2005). LLMs offer a new form of writing support, assisting students in generating ideas, organizing content, and refining their language. Early findings suggest that students who use these tools during the planning and revision phases may produce more coherent and technically accurate texts (Lee, 2023). However, the benefits of LLMs are not automatic. Their effectiveness hinges on how they are introduced and used in educational contexts.

From a TPACK perspective, guided LLM use can enhance academic writing by aligning the tool's features with pedagogical goals. Instructors may, for instance, teach students how to use the model to outline arguments or critically revise text while warning against uncritical copying or overreliance. This structured integration supports metacognitive engagement and allows students to internalize academic writing conventions. In contrast, students in the unguided condition may fail to use the tool optimally. Without pedagogical framing, they might use it only superficially—for grammar correction or idea generation—without fully engaging with the writing process. Additionally, they may be more prone to accept AI-generated suggestions uncritically, leading to errors in reasoning, style, or source use (Wang, 2022).

For students in the control condition, the writing task requires managing all stages of composition without external digital support. While this reflects a traditional academic scenario, it may impose greater cognitive demands and limit writing quality, especially for students lacking confidence or fluency in academic writing (Ayeni et al., 2024). Based on this reasoning, the study hypothesizes that students in the guided LLM condition will produce significantly higher-quality academic writing than those in the unguided and control groups, respectively. These differences are theoretically grounded in the TPACK framework and supported by prior research on instructional scaffolding and technology-mediated writing support.

## 4 Perceived mental health

University students' mental health has become a central concern in global higher education, with consistently high levels of anxiety, stress, and emotional exhaustion reported across diverse national contexts (Granieri et al., 2021). These issues are particularly salient in competitive academic environments, where cognitive demands are high and support structures often limited. Academic writing, in particular, is a cognitively and emotionally taxing task that may exacerbate stress, especially in the absence of timely guidance or feedback.

The Cognitive Load Theory (Sweller, 1988) provides a relevant framework for understanding how instructional conditions affect students' mental wellbeing. According to this theory, cognitive performance is shaped by the interplay of three types of load: intrinsic (task complexity), extraneous (inefficient instructional design), and germane (learning-related processing). Poorly structured tasks tend to increase extraneous load, consuming cognitive resources and contributing to frustration or emotional fatigue (Li et al., 2020).

LLMs, when properly embedded in instruction, can help reduce extraneous cognitive load by automating lower-level processes such as sentence formulation, grammar correction, or even idea generation. However, this benefit is not automatic. Students need pedagogical guidance to understand how to use the tool effectively and ethically, and how to interpret or revise its suggestions. Without such framing, students may misuse the tool, become overwhelmed by its outputs, or develop dependency without comprehension (Park and Ahn, 2024).

In the guided condition, students receive structured instructions on how to use the LLM strategically during the writing process—e.g., to plan text sections, refine transitions, or paraphrase while maintaining academic integrity. This structure is expected to reduce cognitive overload and enhance students' sense of control, which may, in turn, support emotional regulation and perceived well-being. In contrast, the unguided group accesses the tool without clear direction. While they may benefit from its features, they also face the burden of interpreting outputs and deciding when and how to use them. This may increase cognitive load rather than reduce it, particularly for students unfamiliar with AI tools or lacking academic writing experience. Consequently, their perceived mental health may remain unchanged or even be negatively affected.

Finally, students in the control group, without access to any external tool or guidance, must complete the writing task using only their own cognitive and emotional resources. While this mirrors traditional academic practice, it may result in heightened task-related anxiety or emotional exhaustion, especially under time constraints or pressure to perform.

Based on this framework, the present study hypothesizes that students in the guided LLM condition will report significantly better mental well-being than those in the control group, with the unguided group expected to fall somewhere in between. This hypothesis reflects the assumption that instructionally structured technology use, rather than mere access, is the key to supporting psychological outcomes in academic settings.

## 5 Academic engagement

Academic engagement is a multidimensional construct encompassing students' behavioral, emotional, and cognitive investment in learning activities (Fredricks et al., 2004). High levels of engagement have been associated with greater academic achievement, persistence, and satisfaction, particularly in university settings where autonomy and self-regulation are central to success (Wang, 2022). However, engagement is also sensitive to fluctuations in motivation, task design, and perceived support from instructors or institutional structures (Lin, 2024).

A useful framework for understanding the mechanisms that foster engagement is the Self-Determination Theory (SDT), proposed by (Deci and Ryan 2000, Ryan and Deci, 2000). According to SDT, engagement flourishes when learners experience the fulfillment of three basic psychological needs: competence (feeling effective), autonomy (feeling self-directed), and relatedness (feeling connected and supported). Instructional strategies that enhance these dimensions are more likely to result in sustained engagement and intrinsic motivation.

In this context, the use of LLMs has the potential to support academic engagement—but only if implemented thoughtfully. In the guided condition, students receive clear instructions on how to use the tool to improve their writing in ways that foster self-efficacy and control. For example, they may learn to use the model to test different formulations, organize their ideas more efficiently, or revise their text in response to feedback. This structured support not only enhances competence, but also promotes autonomy, as students gain agency in managing complex academic tasks. In contrast, students in the unguided condition are left to navigate the LLM independently. While some may explore the tool productively, others may feel uncertain about how to use it effectively or ethically. This ambiguity can hinder perceived competence and reduce the motivational benefits typically associated with technology-enhanced learning. Without explicit pedagogical framing, LLM use may become a passive or confusing experience, diminishing its capacity to support sustained engagement.

Finally, students in the control group engage in the task without any digital support. Although this may reflect a traditional educational scenario, it offers limited opportunities to enhance autonomy or competence through external scaffolding. For some students, especially those with lower academic confidence, this condition may result in disengagement or surface-level effort. Building on Self-Determination Theory and recent findings on digital learning environments (Wang, 2022), the present study hypothesizes that students in the guided LLM condition will report the highest levels of academic engagement, followed by those in the unguided condition, with the control group expected to exhibit the lowest levels. This hierarchy reflects the assumption that pedagogically structured AI use can enhance both motivation and investment in academic tasks, provided that it supports students' psychological needs.

Taken together, the theoretical and empirical perspectives reviewed above suggest that the impact of AI tools in higher education depends not merely on access to the technology, but critically on how that technology is pedagogically framed and operationalized. While guided use of LLMs has the potential to support students' writing development, reduce extraneous cognitive load, and foster meaningful engagement, unguided use may result in uneven or superficial outcomes. Meanwhile, students who receive no digital support may face greater academic pressure and cognitive effort, particularly when completing complex tasks under time constraints.

To examine these assumptions, the present study adopts an experimental design comparing three conditions: guided LLM use, unguided LLM use, and a control group without access to LLMs. The outcomes under investigation—academic writing quality, perceived mental wellbeing, and academic engagement—were selected because they represent core dimensions of student success and are theoretically linked to instructional design and technological integration. Building on the reviewed literature, it is hypothesized that students in the guided LLM condition will outperform their peers across all three variables, followed by those in the unguided condition, with the control group expected to report the lowest levels of performance and well-being. This hypothesis reflects the view that it is not the technology itself, but rather the pedagogical structuring of its use, that determines its educational value.

### 5.1 Hypotheses

H1: Students in the guided LLM use condition will demonstrate significantly higher academic writing quality than those in the unguided LLM use and control groups.
H2: Students in the guided LLM use condition will report significantly higher levels of perceived mental health compared to students in the control group.
H3: Students in the guided LLM use condition will exhibit significantly greater academic engagement than those in the control group.
H4: Students in the unguided LLM use condition will demonstrate intermediate levels of academic writing quality, perceived mental health, and engagement, higher than those in the control group but lower than those in the guided use group.

## 6 Method

### 6.1 Transparency and openness

In this experimental study, we report how the sample size was determined and all inclusion criteria, manipulations, and outcome measures. All anonymized data are available via the Open Science Framework (https://osf.io/htejm/?view_only=54624dbd9f11467ea26242bae037e713). The data were analyzed using SPSS, version 29. No data were collected after the data analysis began. This study was not preregistered.

### 6.2 Participants

An a priori power analysis was conducted using G^*^Power (Faul et al., 2009) to determine the required sample size for a one-way ANCOVA with three groups and one covariate. Setting the alpha level at 0.05, power at 0.80, and anticipating a small to medium effect size (*f* = _0.20_), the estimated minimum sample size was *N* = 246. The final sample consisted of two hundred and eighty eight undergraduate students enrolled in humanities and arts programs at an international university in China. Instructors from four elective courses were initially contacted via internal mailing lists distributed by the College of Humanities and Arts and were invited to authorize data collection during one of their scheduled sessions. Once instructor consent was obtained, students were approached in person during class and invited to participate. Participants were recruited using a convenience sampling strategy, and participation was strictly voluntary. Students were informed that they could decline or withdraw at any point without penalty. No academic credit, compensation, or incentive was offered. Of the approximately three hundred and twenty five students approached across the four courses, two hundred and eighty eight undergraduate (88.6%) agreed to participate and completed all study components. The final sample included one hundred and sixty eight male students (58.3%) and one hundred and twenty female students (41.7%), ranging in age from 18 to 22 years (*M* = 19.88, *SD* = 1.50). All participants completed the writing task and self-report measures under supervised classroom conditions.

### 6.3 Ethical approval and informed consent

This study was reviewed and approved by the Institutional Review Board (IRB) of the Xi'an International University following the Declaration of Helsinki. Ethical approval was granted on [January 15th, 2024], under the reference number [approval ID, IRB/24/072-HUMARTS]. Before participation, all students received an information sheet outlining the purpose of the study, the nature of the tasks, the voluntary nature of their participation, and their right to withdraw at any point without penalty. Written informed consent was obtained from all participants. The consent form emphasized that participation was anonymous, data would be kept confidential, and results would be used solely for academic research. Participants were also informed that using the LLM was part of an experimental educational intervention and that their course grades would not be affected by their responses or participation.

### 6.4 Procedure

Instructors from four elective undergraduate courses in the humanities and arts were first contacted via internal mailing lists distributed by the College of Humanities and Arts. After receiving their consent to conduct the study during scheduled class time, students were invited in person to participate. The study was introduced at the beginning of the session, and all students were informed that participation was entirely voluntary and that they could withdraw at any time without consequences. Students who agreed to participate provided informed consent and completed the study during a supervised class session. Participants were randomly assigned to one of three experimental conditions: guided LLM use, unguided LLM use, or control. Random assignment was conducted at the individual level within each classroom using a pre-generated randomization list.

All participants were asked to complete the same academic writing task: a critical essay on the topic “The impact of globalization on contemporary culture”, to be written in 45 m using a computer. Participants in the two experimental conditions used OpenAI's GPT-4, accessed via a monitored institutional interface. No alternative platforms or personal devices were permitted. All students interacted with the same LLM under identical interface conditions. On average, participants in the experimental conditions spent between 25 and 35 m actively interacting with the LLM during the task. In the control condition, students received the following prompt: “Write a critical essay on the impact of globalization, using the provided readings. Structure your argument and support it with specific examples.” No access to LLMs or external writing tools was provided. In the unguided LLM use condition, students were given access to GPT-4 and instructed: “You may use the language model (LLM) in any way you find useful to complete your essay.” No additional instructions, training, or support were provided.

In the guided LLM use condition, participants received the following prompt: “Write a critical essay on the impact of globalization. Use the language model (LLM) to help you generate ideas, organize your arguments, and improve clarity. You may use it to explore different perspectives, revise paragraphs, or paraphrase content. Ensure that your essay reflects critical thinking, coherence, and academic style.”

Before beginning the writing task, this group received a brief 10-m in-class orientation delivered by the course instructor, based on a script prepared by the research team. The orientation covered three key elements: how to formulate effective prompts, how to evaluate AI-generated outputs critically, and how to use the tool ethically in academic contexts. After completing the writing task, participants responded to standardized self-report measures assessing perceived mental health, academic engagement, and a short demographic questionnaire. All responses were submitted digitally and anonymized prior to analysis. No pilot study was conducted prior to the implementation of the experiment.

### 6.5 Instruments

All instructions, writing prompts, the manipulation check, and the self-report measures—except for one—were administered in English, in accordance with the instructional language of the international university where the study took place. The only exception was the Utrecht Work Engagement Scale – Student version (UWES-S), which was administered in its validated Chinese version due to its demonstrated psychometric reliability in Chinese undergraduate populations.

### 6.6 Manipulation check

A manipulation check was administered immediately after the writing task to verify participants' adherence to their assigned intervention condition. Participants responded to two closed-ended questions: (1) “Did you use the language model (LLM) while completing the essay?” (Yes/No), and (2) “Were you instructed on how to use the LLM?” (Yes/No). These items allowed the researchers to determine whether participants in the experimental groups used the LLM as intended, and whether participants in the control group refrained from doing so.

Only participants whose responses were fully consistent with their assigned condition were retained for the main analyses. Specifically, inclusion criteria required that participants in the guided condition reported using the LLM with instructions, those in the unguided condition reported using the LLM without instructions, and those in the control condition reported not using the LLM. Participants who did not meet these criteria were excluded from the final dataset. As a result, the final sample included two hundred and forty six participants who successfully passed the manipulation check and were eligible for analysis.

### 6.7 Text quality

The quality of participants' academic writing was assessed using the Grammarly for Education platform (Grammarly, Inc., 2024). After completing the essay, the experimenter uploaded each text under standardized conditions. Grammarly automatically generated a Performance Score, ranging from 0 to 100, which served as the primary indicator of overall text quality. This composite score reflects the extent to which the writing adheres to grammatical norms, clarity, and effective communication, and it can be improved by addressing the platform's suggested revisions. In addition to the performance score, Grammarly provides detailed linguistic metrics, including word count, average word and sentence length, readability score (based on the Flesch scale) (Flesch, 1948), and vocabulary diversity (e.g., proportion of unique and rare words). These secondary indicators were reviewed to contextualize writing complexity and stylistic variation, though only the Performance Score was used in the statistical analyses. This approach provided a replicable, standardized, and objective method for evaluating the quality of written academic work across all participants, minimizing potential biases associated with human ratings.

### 6.8 Mental health

Perceived psychological well-being was assessed using the Ryff Psychological Well-Being Scale (PWBS) (Ryff, 1989). The scale consists of multiple subdimensions (e.g., autonomy, environmental mastery, personal growth, purpose in life), with responses given on a 5-point Likert scale ranging from 1 (strongly disagree) to 5 (strongly agree). Higher scores indicate greater psychological well-being. The PWBS has been widely validated and used across cross-cultural educational contexts (Ryff and Keyes, 1995). Li (2014) tested a shorter version in the Chinese language, and it was used in the present study. In the current sample, internal consistency was acceptable (α = 0.78).

### 6.9 Academic engagement

Academic engagement was measured using the Utrecht Work Engagement Scale—Student Version (UWES-S) (Schaufeli et al., 2002). This 17-item scale captures three core dimensions of engagement—vigor, dedication, and absorption. Participants rated each item on a 7-point scale from 0 (never) to 6 (always). Total scores were calculated by averaging across all items, with higher scores reflecting greater engagement. The UWES-S has demonstrated strong internal consistency and cross-cultural validity (Schaufeli and Bakker, 2004). The Chinese version developed by Fang et al. (2008) was used. In the present study, the scale demonstrated good internal consistency (α = 0.89).

### 6.10 Covariate: prior academic performance

All main analyses included participants' prior academic performance in a literature-related subject as a covariate. Academic records provided a numerical score on a 100-point scale, reflecting performance in the most recent literature course completed before the intervention. This variable was used to control for potential baseline differences in academic ability related to writing, critical reading, and content familiarity. The scores ranged from 18 to 78, with a mean of 44.37 *(SD* = 13.46), indicating moderate variability across the sample. Controlling for this variable allowed for a more accurate estimation of the intervention effects on the outcome measures.

## 7 Results

### 7.1 Descriptive statistics and correlations

Before conducting the main analyses, descriptive statistics and bivariate correlations were calculated for all continuous variables: prior academic performance, text quality, perceived mental health, and academic engagement. Table 1 presents the means and standard deviations for each variable. Pearson's correlation coefficients are presented in Table 2. All correlations were statistically significant at the *p* < 0.01 level. Academic performance was positively correlated with text quality (*r* = 0.258*, p* < 0.001), mental health *(r* = 0.329, *p* < 0.001), and engagement (r = 0.272, *p* < 0.001). Text quality also showed moderate positive correlations with mental health (*r* =0.406, *p* < 0.001) and engagement (*r* = 0.280, *p* < 0.001). The strongest association was observed between mental health and academic engagement (*r* = 0.568, *p* < 0.001), suggesting a meaningful link between students' psychological wellbeing and their engagement with academic tasks.

**Table 1:** Descriptive statistics and pearson correlations between study variables *(N* = 288).

**Variable**	***M***	***SD***	**1**	**2**	**3**	**4**
1. Academic performance	44.37	13.46	—			
2. Text quality	123.74	22.86	.258^**^	—		
3. Mental health	3.82	0.82	.329^**^	.406^**^	—	
4. Academic engagement	13.64	2.86	.272^**^	.280^**^	.568^**^	—

*N* = *288 refers to the total number of participants who completed all measures and were included in the descriptive statistics and Pearson correlations. However, only N* = *246 participants who passed the manipulation check were retained for the main ANCOVA analyses. M* = *mean; SD* = *standard deviation*. ^**^ *p*<*0.01*.

**Table 2:** Estimated marginal means for academic writing quality (controlling for academic performance).

**Intervention**	**Mean**	***SE***	**95% CI lower**	**95% CI upper**
Control	109.61	0.70	108.23	110.99
Unguided LLM use	129.61	0.77	128.10	131.13
Guided LLM use	151.35	0.78	149.82	152.87

*Note*. Means are estimated marginal means adjusted for the covariate (academic performance).

A series of Univariate Analyses of Covariance (ANCOVA) were conducted to examine the effects of intervention condition on three outcome variables: academic writing quality, perceived mental health, and academic engagement. The independent variable was the type of LLM integration (guided use, unguided use, and control), and prior academic performance was included as a covariate in all models.

### 7.2 Academic writing quality

The ANCOVA revealed a significant effect of intervention condition on writing quality, *F*_(2, 253)_ = 789.53, *p* < 0.001, with a very large effect size (*R*^2^ adj = 0.863). This indicates that the intervention condition explained approximately 86% of the variance in writing performance, reflecting a strong and practically meaningful impact of structured LLM integration.

Estimated marginal means showed that students in the guided LLM use condition produced significantly higher quality texts (*M* = 151.35, *SE* = 0.775) than those in the unguided use (*M* = 129.61, *SE* = 0.770) and control groups (*M* = 109.61, *SE* = 0.700), as Table 2 shows.

Effect sizes computed with pooled standard deviations showed a very large difference between the guided LLM use and control groups (Cohen's d = 5.16), a large difference between the guided and unguided groups *(d* = 2.53), and a large difference between the unguided and control groups (*d* = 3.83). These values highlight the strong impact of guided LLM use on writing performance, and confirm that even unguided use resulted in substantially better outcomes compared to no use. All pairwise comparisons were statistically significant after Bonferroni correction (*p* < 0.001), as Table 3 shows.

**Table 3:** Pairwise comparisons—academic writing quality.

**Comparison**	**Mean difference (I–J)**	***SE***	***p***	**95% *CI* Lower**	**95% *CI* upper**
Guided LLM use—control	41.73	1.05	< 0.001	39.20	44.27
Guided LLM use—unguided use	21.73	1.09	< 0.001	19.11	24.36
Unguided LLM use—control	20.00	1.05	< 0.001	17.48	22.52

*SE* = *standard error; CI* = *confidence interval. Pairwise comparisons were adjusted using the* Bonferroni correction.

### 7.3 Perceived mental health

The analysis also showed a significant effect of intervention condition on perceived mental health, *F*_(2, 253)_ = 5.78, *p* = 0.004, with a small to moderate effect size (*R*^2^ adj = 0.097). This suggests that nearly 10% of the variability in self-reported mental wellbeing was attributable to the different LLM conditions, indicating a modest yet meaningful contribution of guided use to students' perceived psychological health. Students in the guided use condition reported significantly higher mental health scores (*M* = 4.15, *SE* = 0.076) than the control group (*M* = 3.81, *SE* = 0.069, *p* = 0.003), as Table 4 shows.

**Table 4:** Estimated marginal means for perceived mental health (controlling for academic performance).

**Intervention**	**Mean**	***SE***	**95% *CI* lower**	**95% *CI* upper**
Control	3.81	0.069	3.67	3.94
Unguided LLM use	3.91	0.076	3.76	4.06
Guided LLM use	4.15	0.076	4.00	4.30

Means are estimated marginal means adjusted for the covariate (academic performance).

As Table 5 shows, the difference between the guided and unguided groups (*M* = 3.91, *SE* = 0.076) approached statistical significance *(p* = 0.070), whereas no significant difference was observed between the unguided and control conditions (*p* = 0.984). Effect size estimates indicated a moderate difference between the guided LLM use and control conditions (Cohen's *d* = 0.53), a small to moderate effect between guided and unguided use (*d* = 0.31), and a negligible effect between unguided use and control (*d* = 0.14). These findings suggest that only structured use of the LLM led to meaningful psychological benefits.

**Table 5:** Pairwise Comparisons – Mental Health.

**Comparison**	**Mean difference (I–J)**	**SE**	***p***	**95% CI Lower**	**95% CI Upper**
Guided LLM use—control	0.35	0.10	.003	0.10	0.59
Guided LLM use—unguided Use	0.24	0.11	0.070	−0.01	0.50
Unguided LLM use—control	0.10	0.10	0.984	−0.35	0.15

SE, standard error; CI, confidence interval. Pairwise comparisons were adjusted using the Bonferroni correction.

### 7.4 Academic engagement

Lastly, the ANCOVA for academic engagement indicated a significant effect of intervention condition, *F*_(2, 253)_ = 6.70, *p* = 0.001, with a modest effect size (*R*^2^ adj = 0.101). This means that around 10% of the variance in engagement was explained by the intervention condition, pointing to a small but practically relevant effect of structured LLM integration on students' involvement in academic activities. Students in the guided LLM use group reported the highest engagement scores (*M* = 14.67, *SE* = 0.304), significantly higher than the control group (*M* = 13.17, *SE* = 0.275, *p* < 0.001), as Table 6 shows.

**Table 6:** Estimated marginal means for academic engagement (controlling for academic performance).

**Intervention**	**Mean**	***SE***	**95% *CI* lower**	**95% CI upper**
Control	13.17	0.28	12.62	13.71
Unguided LLM use	13.74	0.30	13.15	14.34
Guided LLM use	14.67	0.30	14.07	15.27

Means are estimated marginal means adjusted for the covariate (academic performance).

Although the unguided use group (*M* = 13.74, *SE* = 0.302) scored higher than the control group, this difference was not statistically significant *(p* = 0.485), and the difference between the guided and unguided groups was marginal (*p* = 0.093), as Table 7 shows. For engagement, the contrast between guided use and control yielded a moderate effect size (Cohen's d = 0.55), while the effect between guided and unguided use was small to moderate (*d* = 0.32), and the difference between unguided use and control was small (*d* = 0.21). These results indicate that guided integration produced a noticeable improvement in students' involvement, whereas unguided use led to minimal gains.

**Table 7:** Pairwise comparisons—academic engagement.

**Comparison**	**Mean difference (I–J)**	***SE***	***p***	**95% *CI* lower**	**95% *CI* upper**
Guided LLM use—control	1.51	0.41	< 0.001	0.51	2.50
Guided LLM use—unguided use	0.93	0.43	0.093	−0.10	1.96
Unguided LLM use—control	0.58	0.41	0.485	−0.41	1.57

SE, standard error; CI, confidence interval. Pairwise comparisons were adjusted using the Bonferroni correction.

These results suggest that the guided integration of LLMs can significantly enhance students' academic writing and engagement, and may also support improvements in perceived mental health, compared to both unguided use and no use of LLMs.

*Hypothesis 4: Intermediate Outcomes in the Unguided LLM Use Condition*

Hypothesis 4 proposed that students in the unguided LLM use condition would demonstrate intermediate levels of academic writing quality, perceived mental health, and academic engagement, higher than those in the control group but lower than those in the guided use group.

The results provided partial support for this hypothesis. In terms of academic writing quality, the unguided group (*M* = 129.61, *SE* = 0.77) scored significantly higher than the control group (*M* = 109.61, *SE* = 0.70, *p* < 0.001), but significantly lower than the guided group (*M* = 151.35, *SE* = 0.78, *p* < 0.001). These findings confirm the predicted ordinal pattern in this domain.

However, for perceived mental health, the unguided group (*M* = 3.91, *SE* = 0.076) did not differ significantly from the control group (*M* = 3.81, *SE* = 0.069, *p* = 0.984), although it trended lower than the guided group (*M* = 4.15, *SE* = 0.076), with this comparison approaching statistical significance (*p* = 0.070).

Similarly, regarding academic engagement, the unguided group (*M* = 13.74, *SE* = 0.30) did not significantly differ from the control group (*M* = 13.17, *SE* = 0.28, *p* = 0.485). The difference between the unguided and guided conditions (*M* = 14.67, *SE* = 0.30) was marginal *(p* = 0.093).

These results indicate that while the unguided LLM condition yielded intermediate outcomes for academic writing quality consistent with Hypothesis 4, the same pattern was not statistically supported in perceived mental health and academic engagement.

## 8 Discussion

### 8.1 H1: Writing quality enhancement through guided LLM use

The results strongly support Hypothesis 1, demonstrating that students who received structured guidance using LLMs achieved significantly higher academic writing quality than those in both the unguided and control groups. The magnitude of the effect was exceptionally large, underscoring the substantial educational potential of guided LLM integration. This finding aligns with the growing body of evidence suggesting that effective integration of LLMs in academic contexts improves the quality of student output and encourages critical engagement with both the writing process and the technology itself (Cash et al., 2025). The superiority of the guided condition can be interpreted through several converging mechanisms identified in recent research. First, structured frameworks like the Writing Path, which utilize explicit outlines, have been shown to significantly improve text generation quality by aligning outputs with the user's intentions and task-specific goals (Lee et al., 2024). This alignment is particularly important in academic settings, where coherence, argument structure, and adherence to conventions are critical.

Moreover, the results reflect broader findings in human-AI collaborative writing research. Studies on tasks such as headline generation show that users achieve better outcomes when they can guide and selectively refine LLM outputs. This process enhances quality without compromising user agency or perceived authorship (Ding et al., 2023). This suggests that guided LLM use in educational settings may strike a productive balance between automation and student ownership. At a cognitive level, guided use of LLMs appears to support key phases in the writing process, particularly translation and revision. Chakrabarty et al. (2024) found that professional writers benefited most from LLM support during these stages. This insight resonates with our results and further substantiates the utility of guided approaches in educational contexts.

Finally, it is worth noting that the enhanced writing performance observed may not stem solely from the tool's linguistic capabilities, but also from reduced uncertainty and cognitive load due to structured task framing. When students know exactly how to proceed and what is expected of them in using a complex tool like an LLM, their cognitive resources may be more efficiently allocated to higher-order writing concerns, thus improving final output quality.

### 8.2 H2: Guided LLM use and perceived mental health

The findings provide empirical support for Hypothesis 2, indicating that students in the guided LLM use condition reported significantly higher levels of perceived mental health compared to those in the control group. Although the effect size was modest, the statistical significance of the difference underscores the potential of guided LLM integration as a psychologically beneficial educational tool. Notably, the comparison between the guided and unguided groups approached significance, suggesting that guidance in LLM use may play a decisive role in how such tools influence users' well-being. These results are consistent with growing evidence that conversational AI systems can enhance users' subjective mental health experiences when implemented with structured guidance. One contributing factor may be the enhanced user experience associated with anthropomorphically designed systems. For instance, Wu et al. (2024) showed that agents like Sunnie increased users' perceptions of usability and engagement. Such design strategies may foster a more human-like, empathetic interaction, which resonates with students in high-stress academic contexts.

Beyond surface-level interaction quality, systems like VITA have demonstrated that adaptive, behavior-sensitive guidance improves not just perception but also outcomes in mental well-being (Spitale et al., 2025). These systems personalize responses to individual user profiles and evolving needs, features that align well with the nature of guided LLM use in this study. When students receive structured prompts, reflective exercises, or scaffolded interactions from LLMs, the result is improved engagement and potentially heightened psychological support.

The results also echo the broader literature on LLM-based agents such as Replika, which offer on-demand, judgment-free interactions. Ma et al. (2023) highlighted how such interactions help individuals engage in self-reflection and develop confidence. In the present context, the structured engagement with LLMs may serve a similar purpose, providing students with an emotionally neutral space to articulate their thoughts and manage academic stress more effectively. Furthermore, Kumar et al. (2024) noted that AI-guided systems can integrate multiple data sources to detect subtle shifts in mental states and deliver personalized micro-interventions. Although this study did not leverage multimodal inputs, the positive outcome observed in the guided condition suggests that even text-based interventions, when strategically framed, can produce a meaningful uplift in well-being.

At the same time, the non-significant difference between the unguided and control groups raises important questions about the boundary conditions under which LLMs can support mental health. One plausible explanation is that unguided access may generate uncertainty, confusion, or even decision fatigue when students are left to navigate the system without structure. Prior research suggests that the absence of guidance can lead to overwhelming interactions or passive use of the tool, which may fail to produce affective benefits (Zhang et al., 2024; Zhu, 2024). It is possible that psychological support through AI requires not only access but also a sense of clarity, safety, and intentionality—conditions more likely to be fostered in structured interventions. In sum, the significant improvement in perceived mental health among students in the guided condition supports the hypothesis that structured, intentional interaction with LLMs can enhance psychological experiences in academic settings. These findings reinforce the view that LLMs—when deployed thoughtfully—can act as supportive companions in educational environments (Youn and Jin, 2021), particularly when combined with design principles and adaptive features that foster trust, personalization, and emotional safety (Liu et al., 2023). However, the lack of improvement in the unguided condition highlights the importance of pedagogical framing as a necessary condition for translating technological affordances into emotional gains.

### 8.3 H3: Guided LLM use and academic engagement

The results support Hypothesis 3, indicating that students in the guided LLM use condition exhibited significantly greater academic engagement than those in the control group. The ANCOVA revealed a statistically significant effect of condition on engagement scores, with a modest effect size. Students in the guided condition reported the highest levels of engagement, reinforcing the view that structured interaction with LLMs can foster a more involved and focused learning experience. These findings align with a growing body of research highlighting the importance of guidance in shaping students' cognitive and emotional engagement in AI-supported learning environments. In particular, structured guidance during LLM use has been shown to reduce off-task behavior, such as random or superficial queries and the indiscriminate use of AI for answer retrieval (Kumar et al., 2024, 2023). By promoting intentional and reflective engagement, guided LLM interventions encourage students to assume more active roles in their learning processes.

Moreover, research on immersive and AI-integrated educational formats—such as Alternate Reality Games (ARGs) augmented with LLM guidance—has demonstrated that such designs can enhance behavioral engagement, emotional connection, and control beliefs (Cheng et al., 2022; Neary and Schueller, 2018). These findings suggest that when LLMs are embedded in pedagogically grounded frameworks, they can catalyze sustained academic motivation and participation. At the cognitive level, integrating LLMs into virtual teaching assistant roles, such as the Jill Watson system, further supports the idea that guided AI interactions can promote higher-order thinking and intellectual curiosity (Maiti and Goel, 2024). Students in such systems are not merely passive recipients of information but are actively encouraged to formulate and refine complex inquiries, fostering deeper engagement with content.

In contrast, using unguided LLMs favors quick information retrieval over sustained learning. Although such interactions may yield short-term performance gains, they do not appear to generate the same level of student investment or trust in the learning process (Kumar et al., 2025). This may help explain why the guided condition in the present study outperformed both the unguided and control groups regarding engagement. Indeed, the absence of clear instructional framing in the unguided condition may have led to uncertainty about how to use the tool productively, diluting its potential benefits for emotional or behavioral engagement. When students are unsure whether they are using a tool “correctly,” this ambiguity can undermine their sense of efficacy and reduce motivation to persist. Thus, although the results confirmed that guided LLM use fosters greater academic engagement, they also suggest that without supportive structure, LLMs may not reliably elicit active academic involvement. By combining technological capabilities with pedagogical intentionality, these systems offer an interactive and supportive environment that encourages students to actively participate, reflect, and persist in their academic work.

### 8.4 H4: Intermediate outcomes of unguided LLM use

Hypothesis 4 posited that students in the unguided LLM use condition would exhibit intermediate levels of academic writing quality, perceived mental health, and engagement, greater than those in the control group but lower than those in the guided use condition. The results partially supported this hypothesis: while this expected pattern was observed and statistically confirmed in academic writing quality, it was not replicated in perceived mental health or academic engagement.

The writing results suggest that access to LLMs can meaningfully enhance students' output even without structured guidance. The unguided group significantly outperformed the control group, indicating that basic interaction with the tool—through prompts, content generation, or surface-level feedback—was sufficient to raise writing quality. This aligns with prior findings that when used independently, LLMs can offer valuable assistance in planning, drafting, and refining text (Jungherr, 2023; Meyer et al., 2024). Nevertheless, the superior performance of the guided group supports the idea that structured scaffolding, metacognitive prompts, and explicit instruction in tool usage amplify these benefits (Cash et al., 2025; Salimi and Hajinia, 2025).

In contrast, the data did not support the predicted intermediate pattern for perceived mental health. Although the unguided group reported slightly higher scores than the control group, this difference was not statistically significant. One possible explanation is that unguided use, while offering on-demand support, lacks the emotional structure and stress regulation strategies typically embedded in guided implementations. Without clear boundaries or reassurance about responsible usage, students may experience friction, uncertainty, or even anxiety about whether they are “using the tool correctly,” which may counteract any potential gains in psychological well-being (Zhang et al., 2024). In this context, guidance may not only clarify functionality but also serve a regulatory role—normalizing AI integration, reducing confusion, and promoting a sense of support and competence (Zhu, 2024).

A similar pattern emerged with academic engagement. Although the unguided group showed numerically higher engagement than the control group, this difference was not statistically significant. This suggests that, while unguided LLM access may spark curiosity and enable autonomous exploration, it does not consistently produce sustained or deep engagement. One likely reason is that students without instructional scaffolding may remain uncertain about how to engage productively with the tool, leading to hesitant or fragmented interaction. Prior research indicates that without pedagogical framing, students may use LLMs for surface-level information retrieval or task avoidance, limiting the depth of their involvement (Chen and Leitch, 2024). By contrast, guided use has been associated with stronger emotional and cognitive engagement, as students are trained to leverage LLMs in a reflective and goal-oriented manner (Beurer-Kellner et al., 2024; Uchendu et al., 2023).

Altogether, the findings highlight the nuanced role of guidance in realizing the potential of LLMs. While unguided use may yield modest benefits in writing quality, its impact on mental health and engagement appears more limited. The lack of significant differences between the unguided and control groups in two of the three outcome domains suggests that students may underutilize these tools or even encounter friction in their use without structured scaffolding. These results point to the importance of not only providing access to AI tools but also offering appropriate pedagogical frameworks to ensure their effective and psychologically supportive implementation.

### 8.5 Limitations

Despite this experimental design's strengths, several limitations should be acknowledged. First, although random assignment was used to allocate participants across conditions, the study relied on self-report manipulation checks to confirm adherence to the assigned use of LLMs. While this approach ensured consistency between reported and intended use, it may introduce response bias or fail to capture subtle variations in how students interpreted and used the tool within each condition (Roshanaei, 2024). Although technically feasible alternatives such as behavioral logging (e.g., tracking LLM interactions) might offer more objective verification, such data were not collected due to ethical constraints and institutional limitations in access control. Future research could explore ways to integrate such measures in a transparent and privacy-respecting manner.

Second, the study was conducted within a single institutional context and with a relatively homogeneous sample—undergraduate students enrolled in humanities and arts programs at an international university in China. This limits the generalizability of the findings to other educational settings, disciplines, and cultural contexts (Salimi and Hajinia, 2025). In particular, students in STEM fields might interact with LLMs differently not only due to varying levels of digital literacy, but also because of the nature of the writing tasks they face, the disciplinary conventions they follow, and the specific modes of information retrieval their fields require. These differences may influence how beneficial, usable, or trustworthy LLM tools appear in practice.

Third, the primary measure of writing quality relied on the automated Performance Score generated by the Grammarly for Education platform. While this tool offers objectivity and replicability, it prioritizes surface-level features such as grammar, clarity, and lexical variety. As a result, it may not fully capture deeper dimensions of academic writing—such as argumentation structure, critical analysis, originality, synthesis of sources, or adherence to disciplinary conventions—which are essential in evaluating high-level academic work. Human-rated assessments or rubric-based evaluations could complement automated scoring in future studies to provide a more holistic picture of writing quality (Salimi and Hajinia, 2025).

Fourth, although the study included a validated measure of prior academic performance as a covariate, this measure was limited to students' most recent literature course. While this represents a meaningful control, the category of “literature-related subject” remains broad and may encompass varying levels of complexity and assessment standards. Moreover, other potentially relevant factors—such as writing experience in other languages, previous exposure to AI tools, or individual motivation—were not controlled and could have influenced the outcomes (Xu et al., 2025). Fifth, the study used perceived mental health and academic engagement as outcome variables measured through self-report scales. While these instruments are widely validated, self-reported data are subject to social desirability and may not accurately reflect behavioral engagement or psychological functioning (Meyer and Elsweiler, 2025). Future research could enhance robustness by incorporating behavioral (e.g., time-on-task) or physiological (e.g., stress monitoring) indicators to triangulate self-perceptions with observable evidence (Youn and Jin, 2021).

To sum up, the findings should be interpreted with an awareness of existing limitations. For example, while guided LLM use markedly improves performance on structured academic tasks, its efficacy may vary across genres or in more creative domains. Gómez-Rodríguez and Williams (2023) noted that human writers still maintain an advantage in areas such as humor and originality, which are difficult for LLMs to replicate reliably. Therefore, while our results highlight the transformative potential of guided LLM use, they also reinforce the need for human oversight and creative judgment in academic writing.

### 8.6 Practical implications for teaching: structured integration of LLMs in academic instruction

Integrating LLMs into academic writing instruction presents promising opportunities and pressing challenges. The present findings reinforce the importance of structured, guided use of LLMs, particularly in enhancing students' writing quality, academic engagement, and, to a certain extent, their perceived mental well-being. These results carry several practical implications for educators, instructional designers, and policymakers in higher education. Importantly, the implications presented here are directly informed by the limitations discussed above, and their placement after the limitations section reflects a deliberate decision to ensure that recommendations are realistic, context-aware, and attuned to the boundaries of the current design.

### 8.7 Designing guided LLM integration

The study underscores the pedagogical value of structured engagement with LLMs. Educators should prioritize guided frameworks when introducing LLMs into learning environments (Alsobeh and Woodward, 2023). This includes providing students with clear instructions on using these tools effectively, offering structured prompts, and integrating LLM interactions into existing learning goals (Chiang and Lee, 2023). As demonstrated by approaches such as the Writing Path framework (Lee et al., 2024), guided strategies help align LLM-generated content with academic standards and user intentions, improving writing quality (Gómez-Rodríguez and Williams, 2023). Guidance also plays a crucial role in shaping student behavior during AI interaction. Research shows that structured guidance can reduce off-task or random queries and foster a more focused, problem-solving approach to writing (Kulaksiz, 2024). When embedded in pedagogy, these strategies enhance performance and increase students' trust and sense of ownership in the learning process (Bekker, 2024).

### 8.8 The role of educators in mediating LLM use

LLM integration necessitates rediscovering the educator's role—from transmitter of knowledge to AI literacy and ethical engagement facilitator (Lazebnik and Rosenfeld, 2024). Teachers should take an active role in helping students understand the limitations of LLMs, differentiate between responsible use and misuse, and navigate the ethical considerations associated with AI-generated content (Lee, 2023). Training students to critically evaluate and revise LLM outputs contributes to deeper learning and helps prevent overreliance (Liao et al., 2023). Instructors can also promote transparency by encouraging students to document their use of LLMs in the writing process, thus reinforcing principles of academic integrity and accountability (Mahmoud and Sørensen, 2024). This approach fosters a culture of AI-augmented authorship, where students learn to integrate feedback rather than delegate writing tasks to an automated agent. This pedagogical vision also aligns with global policy agendas—such as the UNESCO Futures of Education framework and the Sustainable Development Goal 4 (SDG4)—by promoting inclusive, equitable, and future-ready higher education that incorporates responsible AI use.

### 8.9 Risks of unguided use and the need for policy

While the study found that even unguided use of LLMs can yield some benefits, particularly in writing quality, such benefits are significantly more limited without instructional scaffolding. Unguided use has several risks, including superficial engagement, skill stagnation, and academic integrity concerns. Without explicit instruction, students may bypass the cognitive and metacognitive processes essential to writing, relying instead on the fluency of LLMs to complete tasks (Lopes et al., 2024). Moreover, the indistinguishability of AI-generated text from human-authored work poses significant challenges for evaluation (De Villiers et al., 2024; Reinhart et al., 2024). This complicates the role of assessment and highlights the urgent need for institutional policies that address transparency, disclosure practices, and acceptable uses of generative AI in coursework. As noted in the limitations, behavioral metrics and clearer definitions of disciplinary norms could inform these policies, especially when automated scoring tools like Grammarly are involved.

### 8.10 Fostering independent skill development

Finally, educators must balance leveraging the benefits of LLMs and fostering independent writing skills (Patac and Patac Jr, 2025). While guided use can accelerate learning and reduce barriers, overdependence on AI tools may inhibit students' ability to think critically and write autonomously (Ouwehand et al., 2025). Integrating LLMs should not replace traditional instruction but complement it through strategy-based interventions, peer review, and scaffolded writing tasks (Li et al., 2023; Lin et al., 2024). Future research might also explore how these strategies can be adapted to STEM disciplines or interdisciplinary programs, as differences in writing genres and task complexity may shape how students engage with LLMs. In line with SD4′s commitment to inclusive and contextually sensitive education, such differentiated approaches are essential to ensuring that AI-enhanced instruction serves diverse learners effectively.

## 9 Conclusion

This study provides empirical evidence for the differential effects of guided and unguided integration of LLMs on students' academic writing quality, perceived mental health, and academic engagement in higher education. The findings demonstrate that guided LLM use consistently outperforms both unguided and no use, particularly in improving writing outcomes and fostering student engagement. These effects are most pronounced when LLMs are embedded within structured instructional frameworks that promote critical interaction, strategic thinking, and responsible use.

Notably, unguided LLM use yielded only partial benefits. While it enhanced academic writing quality relative to the control group, it did not significantly improve perceived mental health or engagement. These results suggest that unstructured exposure to generative AI may not be sufficient to produce holistic educational gains. Instead, pedagogical scaffolding and active instructor involvement appear essential to unlock the full potential of LLMs in supporting learning, well-being, and student agency. The study contributes to the growing literature on human–AI collaboration in education by underscoring the importance of designing intentional, ethically informed, and learner-centered approaches to AI integration. As educational institutions increasingly adopt LLM-based tools, the distinction between guided and unguided use becomes pedagogically relevant—because of its impact on learning quality, engagement, and wellbeing—and ethically imperative, as it directly affects student autonomy, academic integrity, and equitable access to meaningful AI-supported education.

Looking ahead, future research should explore how guidance strategies can be tailored to different learning profiles, disciplines, and institutional cultures. Longitudinal and mixed-method designs may further illuminate the evolving relationship between students and AI, providing insights into how LLMs shape academic development. Ultimately, the challenge lies in providing access to powerful technologies and designing meaningful and equitable frameworks for their use—frameworks that preserve learning integrity while embracing innovation. The findings of this study call for a thoughtful and pedagogically grounded integration of LLMs in higher education. When embedded in structured learning environments, guided use can enhance academic outcomes, support student wellbeing, and promote ethical use of AI. To realize these benefits, educators must assume an active role in designing, modeling, and monitoring AI engagement, ensuring that LLMs serve as tools for empowerment rather than substitution. Achieving this vision also depends on ongoing professional development for educators, who must be equipped not only with technical competencies but also with pedagogical strategies to guide students in critically and ethically navigating AI-supported academic tasks.

## 10 Declarations

Ethical approval and Informed Consent for participation: This study was reviewed and approved by the Institutional Review Board (IRB) of Xi'an International University following the Declaration of Helsinki. Ethical approval was granted on [January 15th, 2024], under the reference number [approval ID, IRB/24/072-HUMARTS]. Before participation, all students received an information sheet outlining the purpose of the study, the nature of the tasks, the voluntary nature of their participation, and their right to withdraw at any point without penalty. Written informed consent was obtained from all participants. The consent form emphasized that participation was anonymous, data would be kept confidential, and results would be used solely for academic research. Participants were also informed that using the LLM was part of an experimental educational intervention and that their course grades would not be affected by their responses or participation.

## Data Availability

The datasets presented in this study can be found in online repositories. The names of the repository/repositories and accession number(s) can be found below: https://osf.io/htejm/?view_only=54624dbd9f11467ea26242bae037e713lt;/bgt;.
